# Development of a scale for the evaluation of patients’ rights prerequisites at educational hospitals in Iran: a study using the Delphi technique

**Published:** 2015-11-14

**Authors:** Sanaz Aazami, Mosayeb Mozafari

**Affiliations:** ^1^ Department of Nursing, Faculty of Nursing and Midwifery, Ilam University of Medical Sciences, Ilam, Iran.

**Keywords:** *Patients’ rights*, *Delphi technique*, *questionnaire design*

## Abstract

The patients’ rights status is one of the essential elements in defining norms related to the concept of clinical governance system. In addition, the patients’ rights status is an important index for quality of care offered in the health care system. However, the lack of a coherent instrument makes it difficult to evaluate patients’ rights status in hospitals and clinics. The aim of this study was to develop an instrument for the evaluation of patients’ rights prerequisites at educational hospitals in Iran.

This study was conducted using the modified Delphi technique. In this study, 36 experts in the fields of law, medicine, and professional ethics were participated. The panel of experts participated in 3 rounds. First, experts were asked to judge some pre-identified items, and then, excluded items were judged again in the second round. At the end of the third round, all of the agreed items were included in the final list to form an evaluative scale on practice of patients’ rights.

Experts were asked to judge a total 171 items in 3 rounds. Around 31% (n = 53) of items obtained the panel’s approval to be included in the final version of the scale. The experts’ opinions were collected using face-to-face interviews and electronic email during a 6-month period of data collection from October 2013 to February 2014. This study developed a 53-item scale for evaluation of patients’ rights prerequisites in educational hospitals in Iran. This scale was developed in 7 areas of commitments including university education, research, supervision, process management, physical structure, organizational policy, and human resources management.

This study developed an evaluative scale to assess the practice of patients’ rights in educational hospitals. The items in the final version of this scale were obtained from a consensus of experts and the instrument can be used to evaluate the context and prerequisites for practice of patients’ rights in Iranian educational hospitals.

## Introduction

The promotion of patients’ rights is among the priorities of healthcare providers and is considered as an indicator of the health state in every community (1). In addition, patients’ rights status is an important index for quality of care offered in the health care system. Iranian comprehensive patients’ bill of rights has been framed in 5 chapters and 37 articles including values, visions, and one explanatory note. The topics of these 5 chapters are receiving appropriate care, right to obtain sufficient amount of desired information, right to an unrestricted decision on receiving health-care services, right of privacy and confidentiality, and right to access an efficient complaint system in articles number 14, 4, 7, 9, and 3, respectively (2). The results of previous studies show that realization of patients’ bill of rights requires the provision of commitments, infrastructure, and constructive implementation (3). In other words, existence of such a charter independently does not guarantee development and practice of patient’s right. The World Health Organization (WHO) also believes that the sole existence of the charter will not lead to optimization of patients’ rights. Patients’ rights will be effectively practiced in presence of appropriate policies, collaboration, and public awareness, community empowerment along with development of economic, social, and cultural indices (4). Establishment of the patients' bill of rights content entails several important prerequisites which can be achieved by Intrasectional and intersectional collaboration (5). Therefore, some experts and researchers believe that developing instruments to evaluate adherence to patients’ rights is one of the prerequisites for observing the contents of patients’ bill of rights (2). However, lack of a coherent instrument makes it difficult to evaluate patients’ rights status and it is not enough to rely on the content of the patients’ bill of rights for developing such instruments. In addition, optimizing patients’ bill of rights requires the preparation of preconditions in several fields such as management of organization, structure, human resource, and organizational process. Therefore, it is necessary to consider not only the content of the patients’ bill of rights, but also, the mentioned preconditions before developing an instrument to evaluate adherence to the patients’ bill of rights.

We were not able to find a valid and reliable instrument which can assess observance to patients’ rights in Iran. Furthermore, patients’ rights are important indices in the clinical governance system, and organizational excellence and quality improvement which encouraged us to develop a questionnaire for assessment of patients’ rights in Iran. Thereby, we designed this study in two stages. The first stage included assessment of the commitments for establishing patients’ rights in Iranian educational hospitals which has been published elsewhere (3). Within the first stage, 7 areas of commitments were recognized using the Delphi technique including university education, research, supervision, processes management, physical structure, organizational policy, and management of human resource. In addition, each and every area was composed of several administrative recommendations which are directed toward establishment and maintenance of patient rights. 

Within the second stage, which is the current study, authors developed a scale with 7 subscales for evaluating the requirements of patients’ rights practice at educational hospitals in Iran. The scale was developed based on the 7 recognized areas and experts’ opinions in each area. 

**Delphi technique**

The Delphi technique is a subjective-intuitive systematic method of forecasting for collating judgments from a panel of experts on a subject (6). The RAND Corporation (Santa Monica, California, USA) developed the Delphi technique in the 1950’s during an operational research. The Delphi technique gathers the knowledge of experts on a subject during a structured group communication process in which decisions are made in several rounds until a consensus emerges (7-9). Common surveys try to answer “what is?”, whereas, the Delphi technique attempts to address “what could/ should be?” (8). Despite several modifications in the Delphi technique, it is still a valuable method of collating expert’s judgments using an interactive structured process which eventually leads to consensus on a subject. The aim of this technique is to achieve consensus on a subject among a panel of experts in which a series of anonymous questionnaires is administrated in several rounds and feedback is obtained in each round (10). Homogeneous and independent experts are asked to professionally judge a special topic over a wide geographical area using a questionnaire administrated in several rounds and continued until consensus emerges (6-10). The results of each round is reviewed and edited in the next round. There are several benefits to the Delphi technique which result in the prioritization of this method over focused group approaches. Factors which affect the expert’s opinion in the focused group approaches consist of personal interaction of experts, time limitation, and group dynamics and needs. However, in the Delphi technique, there are no time and location limitations, experts are blinded to each other’s identity, and therefore, the experts face no limitation in expressing their opinions. Furthermore, the results derived from the Delphi technique can be interpreted using statistical methods (8, 9, 11). 

## Methods

This study was conducted using the modified Delphi technique in which data were collected through face-to-face interviews and electronic mail during a period of 6 months from October 2013 to March 2014. The Ethics Committee of the Ilam University of Medical sciences, Iran, approved this study. 

The present study was performed in 3 rounds of the Delphi technique. Initially, we prepared appropriate questions for this study. The main question in this study was: “what should be placed in an evaluative scale to measure practice of the patients’ rights?” In the next step, the primary version of the scale was developed in 7 areas based on the results derived from the first stage (3). 

The primary version of the scale was evaluated and edited by 5 experts (3 experts in medical ethics, 1 physician who was head of a hospital, and 1 nurse who conduct research in patients’ right issues). Corrections suggested by experts were applied and content of the scale were revised and prepared for the first round of the Delphi technique with 95 items. Finally, experts were identified and included in the panel of experts based on their qualifications and experiences in the relevant scientific and academic fields. The initial list of experts was composed of 45 professionals. It included 6 PhD holders in the field of management, particularly quality management, 9 in ethics and medical ethics, 6 in Islamic ethics (clergy men), and 8 clinical specialists who were at least assistant professors. This list also included 5 clinical nurses who had worked for at least 15 years, 8 managers and heads of medium and large hospitals with more than 300 beds (4 physicians and the rest non-physicians), and 3 hospital architects.

Upon identifying the panel of experts, we sent an invitation letter along with a prepared booklet to each of them. The prepared booklet contained a summary of the Delphi technique, patients’ bill of rights, and objectives of the current study. Out of the 45 invited experts, 3 refused to participate in the study and we did not receive any response from 6 of them. Therefore, 36 professions (15 females and 21 males) were included in our study. The panelists were informed that this study included 3 rounds and they are required to participate in all of the rounds. The panelists were asked to rate each version of the scale on a 7-item Likert type scale ranging from 1 (strongly agree) to 7 (strongly disagree). 

Furthermore, the opinion of the panelists was asked for any correction, deletion, or suggestion of a new version of the items. Upon receiving all of the comments from the first round, we calculated mean, frequency, and percentage of agreement on each version. Then, we constructed the new version of the scale according to the agreement rate and suggestions from experts. This new version of the scale was sent to the experts along with necessary requirements. We asked panelists to rate the scale again and this continued until the third round in which we finalized the scale according to the established criteria. 

Regarding the instruction to approve an item, it should be noted that experts reported their agreement in answers ranging from 1 to 7 representing strongly agree, moderately agree, somewhat agree, neutral, somewhat disagree, moderately disagree, and strongly disagree. The criteria for approving an item was obtaining at least 70% response rate of “strongly agree” by the experts. On the other hand, the criteria to reject the item was obtaining at least 70% response rate of “strongly disagree” by experts. Eventually, in the case that responses fall between these two criteria, the item is labeled as not approved and is included in the next round of the Delphi method. This process will continue until a consensus is reached on approval or denial of an item. This approach has been frequently used in the Delphi method studies to decrease risk of low level of agreement between experts (6-11). This study was conducted through face-to-face interviews and electronic mail. 

**Scoring**

The scale was scored using the scoring system of the European Foundation for Quality Management (EFQM) excellent model (named RADAR logic) (12). This is due to the fact that we aim to develop a scale for assessing patients’ rights practice in health care organizations. Besides, the EFQM model has widely been used by Iranian researchers (13). This study was scored according to the RADAR (results, approach, deployment, assessment, and review) method of the EFQM excellent model. Approach means what an organization plans to reach and the reason it implies. Deployment consists of 2 parts of approach implementation and systematic implementation of the approaches. Implementation refers to carrying out and practice of the plan and systematic utilization of the approach. Assessment and review imply approach measurements, learning, and improvement of their performance. Measurement includes regular assessment of the effectiveness of this approach and building it through focusing on technique rather than on quantitative values. Training refers to the training activities performed in order to identify and use the best practices within and outside the organization. Improvement is the outcome expected from measurement and training. Therefore, these two activities can be used in identifying, prioritizing, planning, and implementing improvements (14). 

Each of the RADAR elements obtained a score between 0-100% in every round of the study and the average score obtained from the 3 rounds composed the overall score of each element. 

Principles and guidelines for the degree of evidences are described in the EFQM model (13) which has been used in several studies (11, 12). The evidences are categorized based on the model guidelines that contain 5 classifications. These classifications are comprehensive evidence (86-100%), clear evidence (61-85%), good evidence (36-60%), little evidence (11-35%), and lack of evidence (0-10%).

## Results

All of the 36 experts that agreed to participate in the current study were engaged in the 3 rounds of this study. The experts in this study consisted of 15 females and 21 males. In terms of educational attainments, 15 of them hold PhD certificates; 5 in management (particularly quality management), and 4 in nursing, and 6 were clinical specialists. Moreover, 7 of them were specialists in ethics and medical ethics, 5 were clergy men and experts in Islamic ethics, 7 were managers of medium and large hospitals (4 physicians and the rest non-physicians), and 2 were architects.

Tables 1 and 2 show the detail of the performance of the 3 rounds of the Delphi technique. Within the first round, experts strongly agreed on 33 items out of 95 which were included in the final version of the scale. In addition, there were 25 items which received suggestions and comments mainly from research and organizational fields and to a lower degree from human resources and provisional fields. Some of the suggestions and comments include edition of items, grammar editing and revising the content. Thus, 5 items where added and 25 were modified which resulted in the inclusion of 46 items in the second round. However, after applying corrections received from the experts, an overlap was observed between 2 items. Therefore, one of them was excluded from the study and a total 45 items were included in the second round. 

**Table1:** Results of round 1 to 3 of the Delphi technique

**Rounds Criterion**		**Education**	**Research**	**Processes management**	**Provision**	**Organizational policy**	**Physical structures**	**Human resources**	**Total**
**First ** **round**	Items entered into 1^st^ round	15	18	21	9	10	10	12	95
	Approved items	5	4	6	5	3	6	4	33
	Disapproves items	3	4	3	2	1	1	2	16
	Modified items	4	5	4	2	5	3	2	25
	Added items	1	0	1	0	1	0	2	5
	Rejected items	3	5	8	0	1	0	4	21
	**Items entered into 2** ^nd^ ** round**	7	9	8	4	7	4	6	45
**Second round**	Approved items	2	2	3	1	2	0	1	11
	Disapproved items	3	5	3	2	2	2	2	19
	Modified items	1	2	1	1	2	1	2	10
	Added items	0	0	0	0	0	1	1	2
	Rejected items	1	0	1	0	1	1	1	5
	**Items entered into 3** ^rd^ ** round**	4	7	4	3	4	4	5	31
**Third round**	Approved items	1	1	2	0	0	1	2	9
	Disapproved items	3	6	2	1	4	3	3	22
**Final scale**		8	7	11	8	5	7	7	53

**Table 2:** Agreement percentage in the first and final rounds on suggested phrases

**Criterion**	**Approved items in ** **the 1** ^st^ ** round (%)**	**Approved items in ** **all three rounds (%)**
**Human resources**	33.0	30.5
**Physical structures**	60.0	38.9
**Organizational policy**	30.0	23.8
**Provision**	55.6	50.0
**Process management**	28.6	33.4
**Research**	22.3	20.6
**Education**	33.0	30.8
**Total**	34.73	31.0

Within the second round of this study, 45 items were judged by the experts, 11 of which obtained at least 70% agreement and were included in the final version of the scale. In this round, 2 items were added by experts, 10 items were edited, 5 items were rejected, and 19 items did not obtain the minimum agreement rate and were included in the third round for further investigation. 

Out of the 31 items that were included in the third round, only 9 items obtained the minimum agreement rate. Finally, the scale for evaluation of patients' rights prerequisite in educational hospitals was developed with a total of 53 items (Attachment 1).

## Discussion

This study was aimed at developing a Persian version of a scale for evaluation of patients’ rights prerequisite using the opinion and judgment of experts. To the best of our knowledge, the developed scale in this study is the first scale exclusively designed to measure patients’ rights observance in Iranian educational hospitals based on experts’ opinions.

Albeit, we are aware of one existing Persian questionnaire for measuring patients’ rights observance (15), but it has some shortcomings. For example, this scale is general, is mixed with other areas (e.g. clinical governance), and the scoring depends on the evaluator’s interpretation. This questionnaire is part of a book on accreditation of hospitals and clinical governance-related issues. The items are scattered in different parts of this book and are not able to capture a clear profile of patients’ rights and infrastructure in a hospital. In addition, the literature does not show any evidence of using this questionnaire as an instrument to measure patients’ rights. There are also some scales which have been constructed based on physicians’, nurses’, and patients’ points of view (16-18) and no organizational requirement has been considered in such questionnaires. These limitations illustrate that we must elaborate the discussion by showing that criterion and items in the scale are reflective of prerequisites for patients’ rights provision. The scale that we developed in this study has an important feature that distinguishes it from other scales. This is due to the fact that we employed an established approach to developing this scale. The approach has been adopted from the EFQM excellent model and guarantees that the obtained score is a function of the established and continuous processes in organizations (12). 

The results of our study showed that the developed scale was composed of 53 items in 7 areas including education, research, provision, process management, physical structure, organizational policy, and human resource management. Assessment of the items in the final version of our developed scale revealed that consensus was reached among experts on issues that play an important role in patients’ rights. Some of these issues are formation of an ethical decision-making committee (second article of organizational policy subscale), ethical committee in research, and dissemination of ethical consideration among researchers (research items), assessment of violence cases, complaint processing system, and timely response from the patients’ complaint processing system (item 1, 7, and 8 from the provision subscale). 

Sarbaz and Kimiafar (19) have suggested that supervisors and technical assistants in hospitals may control the practice of patients’ rights (19). In addition, Kagoya et al (20) recommended the formation of a governance committee in hospitals to control the observance of patients’ rights (20). Establishment of an informal committee to voice patients’ complaints in educational hospitals enables matters to be resolved immediately while avoiding the need for litigation and official disciplinary processes. Moreover, these informal committees could rely on ethical approaches to resolve patients’ complaints rather than formal disciplinary proceedings (21). The decisions and advices of commissioners’ are remarkably important for health professionals in some countries such as New Zealand (22). Among health care professionals and even the public in New Zealand, commissioner’s decisions outweigh judiciary resolutions (23). Moreover, reports from the province of Quebec, Canada, have shown that more than 98% of health care professionals accept decisions made by informal committees (24). Successful use of the informal committee has been reported in several countries such as Norway, United Kingdom, Finland, and Hungary (25-28). In Germany, at least 149 clinical ethics committees and 86 behavioral counseling institutions have been established to maintain patients’ right practice in more than 77 hospitals since 2005 (29). In 2007, another 29 educational hospitals were added to the mentioned list (30). 

These evidences show that the role of informal committees in resolving patients’ complaints could be less expedient and systematic in comparison to formal multidisciplinary proceedings. Non-judiciary and fair treatment of patients’ complaints may facilitate the acceptance of informal committees’ decisions by patients and caregivers. Furthermore, Parsapour et al. recommended establishing an organized patient compliant system in health centers as a way to improve patients' rights status (31). In order to improve the function of informal committees in regulation of health care services, it is necessary to provide more information and latitude for the committees. Additionally, it would be beneficial to introduce and enforce patients’ rights to health care institutions, public, and governments (26). 

With regards to the educational area, there was a consensus among professionals on the need for introduction of patients’ rights in orientation meetings of new staff, continuity of education among staff, revision and development of a patient-centered syllabus, and evaluation of students according to their adherence to patients’ bill of rights. Sarbaz and Kimiafar (19) considered education as a way to affect health care professionals’ attitudes and eventually increase their adherence to patients’ rights (19). These authors also believed that patients’ rights should be the main focus of medical education along with teaching support and communication skills to students. Continuous education programs, patient education, and developing protocols for observing patients' rights are suggested for the establishment of patients' rights practice in health centers. Parsapour et al. revealed that supplementary education is needed to inform health-care workers about patients’ rights issues, particularly, patients’ access to clinical information and decision-making (31). According to results of the qualitative study by Kagoya et al (20) patients’ knowledge about their rights was correlated with employment status, educational level, and number of referrals to hospital (20). In addition, knowledge of staff about patients' rights correlated with educational level and years of experience as a nurse (20). Our study showed a consensus among panelists about the role of public education on patients’ awareness about their own rights. These findings are consistent with that of the studies by Dadkhah et al. (32) and Ansari et al. (33) that showed a positive significant correlation between educational level and patients’ knowledge about their own rights. These researchers believe that media can play an important role in promoting public sensitivity to patients' rights issues. According to the majority of researches, patients with higher educational level have more expectations regarding their rights (1). In this vein, Kagoya et al (20) suggested the establishment of a patients' rights guideline in hospitals and other health care organizations, mass media awareness about patient rights, and legal advocacy for patients who suffered from violence (20).

The current study showed that criteria such as human resources, process management, and physical aspects of organization are other issues that obtained consensus among panelists. These key areas are the main aspects of any organization especially health care organizations. Kagoya et al (20) revealed that organizational barriers to practicing patients’ rights are financial constraint, shortage of the human resources such as physicians and nurses, lack of communication channels to spread information on patients’ rights, and lack of communication skills (20). Furthermore, Kagoya et al (20) found that 81.5% of patients and 69.4% of health workers were not aware of the patients’ rights charter in Uganda (20). They found that 36.5% of patients experience violation of their right, while 79% of them never demand their own right (20). In addition, the results of the study by Ducinskiene et al (34) in Litiwani showed that 56% of patients were aware of their own rights (34). Bǘken and Bǘken (35) revealed that infrastructure and management problems are the most important causes of lack of appropriate patients’ rights practice in Turkey . 

Urlich et al (36) assessed 600 studies that link the characteristics of the physical environment of hospitals to patient and staff outcomes . Their findings showed that there is a significant linkage between physical environment and several outcomes including staff stress and effectiveness, patient and family stress, effectiveness in delivering care, patient safety, quality of care, and reducing costs (36). These outcomes are representative of human and patients’ rights. The authors emphasized the use of evidence-based practice in designing hospitals to ensure patients’ rights practice. An Italian study investigated spatial factors in designing hospitals which influence the right to health (37). The mentioned Italian study indicated that spatial configuration of hospital spaces influence behavioral model of communication and relationship between patients and health-care staff which, evidently, are the key elements in the right to health. The authors declare that studying spatial configuration in hospitals is an effective instrument for policy makers in decision-making toward increasing quality of care and provision of patients’ rights. There are several elements in designing a hospital including easy access to spaces, location of wards, and horizontal linkage between cores, entrance, and connection areas. Among the spatial factors, the integration core of the public and staff are the two most important cores. Furthermore, it has been shown that patients’ privacy and dignity is not respected due to lack of adequate physical space especially in critical care units, staff’s low awareness of patients’ rights, shortage of same-gender care providers (38), and infrastructure limitations (39). 

These findings show that practice of patients’ rights depends on organizations and their resources including human resources and other infrastructures. Therefore, improvement of organizational resources provides an avenue for better practice of patients’ rights. 

The results of the current study show that experts’ decisions were directed toward those main elements that play an important role in maintenance and practice of patient's rights. Basic actions such as resolution of patients’ bill of rights, development of measurement instruments, and awareness of the public and health care workers are required to effectively practice patients’ rights. Awareness of patients of their own rights can lead to some advantages. Examples of these advantages include increased quality of health care services, decreased costs, prompt recovery, decreased length of stay in hospitals, lower risk of physical and spiritual damages, and more importantly, increased dignity of patients through their participation in decision-making (40). The concept of “human rights in patient care” has been extended and referred to human rights principles in the provision of care (41). Applying human rights in the process of patient care requires several measures including policies, laws, course of action to protect human rights in the process of care, and education of health-care providers and patients on human rights principles (41). 

The most helpful intervention for promoting these rights would be to inform people, alongside educating healthcare providers, about patients’ rights issues (1). Moreover, establishment of informal committees in hospitals is necessary to voice patients’ complaints and to effectively practice patients’ rights. This is to ensure patients demand their own rights even in the presence of some barriers including low education, language differences, low socio-economic status, feeling of inferiority toward health workers, and tendency to receive free services (26, 27). On the other hand, the main focus of hospitals is to fulfill patients’ needs rather than promote patients’ rights. In addition, human resources shortage and time limitations restrict the extent of information shared between health care providers and patients. This is in contradiction of professionals’ principles. Therefore, it is necessary to observe the practice of patients’ rights through informal committees that are independent of hospitals (24, 27). 

## Conclusion

In conclusion, our study developed an evaluative scale to assess the practice of patients' rights in which the scoring method was adopted based on the Excellent Fundation Quality Management (EFQM) model. This scale was constructed as a result of consensus that emerged among experts in the field of medical ethics in the Iranian health system. Therefore, this scale can be used as a valid instrument to evaluate prerequisites of patients’ rights and process in Iranian educational hospitals. Furthermore, hospital managers and stockholders may also benefit from this scale by assessing the status of patients’ rights prerequisites and apply the findings.

**Attachment 1:** Patients’ rights prerequisite Scale*

**Items**	**Approach**	**Implementation**	**Evaluation**
	**0-100%**		
**Education**			
1- Is there a compiled program for professors teaching patient rights?			
2- Have the issues related to patient rights been included in the orientation program for new staff?			
3- Is there a continuous education program on patient rights in the organizational setting?			
4- Has the patient rights module been included in the curriculum of medical schools?			
5- Had the revised curriculum been based on patient rights?			
6- Have the activities for improving knowledge, attitude, and practice of students about patient rights been anticipated in the revised curriculum?			
7- Is there any evidence regarding efforts for public education?			
8- Does clinical evaluation of students emphasize practicing patient rights?			
**Research**			
1- Has the research ethics committee been established at the organizational level?			
2- Is there an established policy in research to ensure patient rights are respected?			
3- Are researchers being notified about instructions on protecting confidentiality of the participants’ information?			
4- Are the researchers being notified about instructions on how to maintain the physical privacy of the study participants?			
5- Does the ethics committee inform researchers on the codified legislation designed for human studies?			
6- Are the researchers being notified about instruction on maintaining the participants' authority in decision making?			
7- Are the researchers being notified about instructions on how to avoid conflict of interest in the study?			
**Process Management**			
1- Have the processes of patient care been clearly described?			
2- Have the processes of patient care been exposed to patients?			
3- Do health care providers fulfill patient care according to the described processes?			
4- Is the confidentiality of patient information maintained during the process of admission, treatment, care, and discharge?			
5- Are the key processes of patient care performed according to the standards and the codified legislations (e.g. the patient rights charter)?			
-Consent form			
-Acquittal form			
-Scheduling appointments			
-Complaints			
-Account clearance			
-Delivering the medical record summary to patients			
-Admission			
-Discharge			
-Patient's discharge with personal written consent form			
-Dying patient			
6- Is the patient rights charter recognized at the organizational level?			
7- Are patients informed of the outlines of the patient rights charter upon their admission?			
8- Is there a central unit to facilitate the patients' communication?			
9- Do health care providers have an identity card?			
10- Is there a system for monitoring and regular evaluation of patient's satisfaction?			
11- Is the patient visiting system performed regularly?			
**Items**	**Approach**	**Implementation**	**Evaluation**
**Policy**			
1- Does the strategic plan comprise the issues related to patient rights?			
2- Have the ethics committees been established and deployed?			
3- Has the compliance with Shariah law been formally established?			
4- Has the unit for quality improvement been established in the centers?			
5-Do the centers comply with the decisions made by the quality improvement unit?			
**Human resource**			
1- Is there any evidence on educating patient rights in human resources development programs?			
2- Is there a coherent program for improving staff’s communication skills?			
3- Is the ratio of staff to patient enough?			
4- Does the staff evaluation system consider issues related to patient rights?			
5- Is there a compiled program to ensure that promotional opportunities are equally distributed?			
6- Is there a system for evaluation of staff satisfaction?			
7- Is there a program to teach clinical staff how to make an ethical decision?			
**Physical environment**			
1- Are the diagnosis and curative procedures performed at the standard settings?			
2-Are the toilets run at the defined standard levels?			
3- Are the buildings easily accessed via the public transport system?			
4- Are the buildings easily accessed by children, elderly, pregnant ladies, and handicapped individuals?			
5-Are the signs adequate and illustrative?			
6-Are the centers equipped with facilities such as bank, ATM, or cafeteria?			
7-Has the caring environment been designed to maintain clients' privacy?			
**Provision**			
1- Are there appropriate channels to contact top level managers (suggestion box, contact line, internet, and etc.)?			
2- Is there a system for regular evaluation and monitoring of patient satisfaction?			
3- Does the ethics committee constantly and regularly monitor human studies?			
4- Is there a system for evaluation and improvement of processes in patient care?			
5- Is there regular report and record on actions maintaining patient rights observance?			
6- Does the committee regarding patient issues (e.g. infection control, morbidity, and mortality) hold meetings regularly?			
7- Is there any deadline for responses to patients' complaints?			
8- Is there a system to track and assess violence in hospitals?			

* The original version of this questionnaire was developed in the Persian language. The current English version was translated using backward-forward translation by panels of bilingual experts. Authors who are interested in using the Persian version of this scale may contact the corresponding Author.
